# Forecasting Air Quality in Taiwan by Using Machine Learning

**DOI:** 10.1038/s41598-020-61151-7

**Published:** 2020-03-05

**Authors:** Mike Lee, Larry Lin, Chih-Yuan Chen, Yu Tsao, Ting-Hsuan Yao, Min-Han Fei, Shih-Hau Fang

**Affiliations:** 1 Department of Electrical Engineering, Yuan Ze University, Taoyuan City, Taiwan; 2 MOST Joint Research Center for AI Technology and All Vista Healthcare, Taipei, Taiwan; 3 Far Eastern Group, Taipei, Taiwan; 4 Department of Geography, Chinese Culture University, Taipei, Taiwan; 5 Research Center for Information Technology Innovation, Academia Sinica, Taipei, Taiwan

**Keywords:** Environmental monitoring, Environmental impact

## Abstract

This study proposes a gradient-boosting-based machine learning approach for predicting the PM_2.5_ concentration in Taiwan. The proposed mechanism is evaluated on a large-scale database built by the Environmental Protection Administration, and Central Weather Bureau, Taiwan, which includes data from 77 air monitoring stations and 580 weather stations performing hourly measurements over 1 year. By learning from past records of PM_2.5_ and neighboring weather stations’ climatic information, the forecasting model works well for 24-h prediction at most air stations. This study also investigates the geographical and meteorological divergence for the forecasting results of seven regional monitoring areas. We also compare the prediction performance between Taiwan, Taipei, and London; analyze the impact of industrial pollution; and propose an enhanced version of the prediction model to improve the prediction accuracy. The results indicate that Taipei and London have similar prediction results because these two cities have similar topography (basin) and are financial centers without domestic pollution sources. The results also suggest that after considering industrial impacts by incorporating additional features from the Taichung and Thong-Siau power plants, the proposed method achieves significant improvement in the coefficient of determination (*R*^2^) from 0.58 to 0.71. Moreover, for Taichung City the root-mean-square error decreases from 8.56 for the conventional approach to 7.06 for the proposed method.

## Introduction

The importance of air quality prediction has increased rapidly for providing citizens with superior quality of life and for enabling effective environmental sensing research. Numerous studies have linked PM_2.5_ (particulate matter) to health problems. For example, previous studies have indicated that poor air quality potentially causes various health problems, including stroke, ischemic heart disease, lung cancer, respiratory infection, and chronic obstructive pulmonary disease^1,2^. Thus, air quality has recently become one of the most important targets of national environmental monitoring and forecasting tasks for many countries in Europe, North America, and Asia^3^. For example, authorities in London cooperate with an environmental research group that provides data for independent scientific studies. The United States Environmental Protection Agency provides raw data, statistics, and visualization for the same purpose^4,5^. In Asia, Taiwan’s Environmental Protection Administration (EPA) set up 19 weather stations and began to monitor the air quality in major cities in 1980. In 1993, the Taiwanese government developed the Taiwan Air Quality Monitoring Network, which includes a large-scale deployment of air monitoring stations, to record and forecast the air quality^6^. This network has been operating for over 25 years and its data are open to the public. Apart from the Taiwanese government, the Taiwanese national research institution Academia Sinica partnered with private industry and civil communities to initiate the Air Box collaborative project to monitor the PM_2.5_ concentration^3,7–9^ by using a large number of small devices. This project has made collecting PM_2.5_ data more convenient and made independent research easier. However, PM_2.5_ values from the Air Box project have low correlation coefficients with the EPA data. Only a moderate positive relationship is observed between them^10,11^, and the reliability of these data is challenged owing to the lack of hardware calibration. Consequently, this study uses the reliable large-scale data collected from the EPA to develop an accurate air quality forecast model for citizens in Taiwan.

Physical models and machine learning models are two types of techniques to forecast the air quality. In the 1990s, various atmospheric dynamics methods were applied to build a physical model with complicated equations^12–17^. These methods required high-speed computers with large memories to calculate a large number of iterations. They had limited accuracy and could not identify the importance of new and old data^18^. Artificial intelligence has recently attracted considerable attention, and various machine learning approaches have been extensively implemented to model data in numerous applications. Air quality forecasting is one promising application. For example, an artificial neural network was applied to forecast air pollutants in Athens, Greece, in 1999^19^. Another study applied a genetic algorithm to air quality prediction for extracting sufficient features and successfully predicted the air quality in Tianjin, China, in 2011^20^. Previous studies have indicated that with the massive collection of data and development of machine learning techniques, a detailed air quality prediction model is worth further study^7,8^. Among various machine learning algorithms, tree-based methods have attracted interest for air quality prediction. A study conducted in 2010 indicated that a decision tree exhibits the best performance when predicting CO_2_ concentrations in Japan^21^. In 2016, a random forest model was applied to predict the air quality in Shenyang, China. A recent study successfully predicted the air quality index with high precision by combining urban sensing data including meteorology, road information, the real-time traffic status, and the points of interest distribution^22^. Specifically, the tree-based learning models generally provide the highest accuracy^23,24^. Besides, in recent years, the tree-based models are also applied for prediction of nitrogen oxides and ozone with high accuracy^25,26^. Therefore, this study selected a gradient boosting model (GBM) on decision trees to generate an air quality forecasting model in Taiwan.

This paper has three main contributions,

First, this study extracted the temporal sequences of highly dimensional and heterogeneous sensor data from both air monitoring and weather stations into a feature domain. The proposed mechanism based on the gradient boosting algorithm and extracted features efficiently predicted the air quality PM_2.5_ for the next 24 h at individual air monitoring stations. Through experiments conducted over 1 year, this study investigated the geographical and meteorological divergence of forecasting results and performed measurements at seven regional monitoring areas in Taiwan.

Second, the prediction performance between Taiwan, Taipei, and London was compared under the same methodology. The results indicate that Taiwan has a more complex environment than London does due to domestic and overseas (mainly from China) sources of PM_2.5_ and the geographic topography of Taiwan. An interesting observation is that the performance for Taipei is similar to that for London, with a stationary prediction performance of approximately 6 in terms of the root-mean-square error (RMSE) and 0.75 in terms of the coefficient of determination (R^2^). These results may be explained by the fact that these two cities have similar topography (basin) and are financial centers without domestic pollution sources. The results from an alternative London database successfully verified the proposed prediction mechanism.

Third, we focused on central Taiwan, especially Taichung City, because air pollution has worsened there in recent years. The main reason for this change is the fact that the second-largest coal-fired power station in the world (i.e., the Taichung Power Plant) is located in this region. This domestic industrial source makes prediction difficult. Thus, we propose an enhanced fusion version of the prediction model by incorporating additional features from Continuous Emission Monitoring Systems (CEMS) to improve the prediction accuracy. After considering the industrial impact, the proposed method achieved significant improvement on average for R^2^ (0.58 to 0.71) and reduced the RMSE (8.56 to 7.06) compared with the conventional approach followed in Taichung City. To the best of our knowledge, this study is the first to combining the use of both air station and industry records for air quality prediction in complex environments.

## Geographical and Meteorological Divergence of PM_2.5_ Concentration Between Regional Monitoring Areas

In Taiwan, the PM_2.5_ concentration has been a severe environmental problem and has attracted considerable attention from researchers and the general public. Previous studies have focused on the source contributions of air pollution. Air pollution can be categorized as locally produced(mainly from motor vehicles and power plants) or long-range transported (LRT) pollution (mainly from China)^27,28^. Some studies have focused on the influence of meteorological conditions on the PM_2.5_ concentration and have indicated that the PM_2.5_ concentration is strongly influenced by the southwesterly monsoonal flow (SWM) and northeasterly monsoonal flow (NEM). Geographical divergence is an important factor when discussing the effects of meteorological conditions and synoptic weather on the air quality in Taiwan^29^. Thus, the following section discusses the geographical divergence and temporal variations of the measured PM_2.5_ concentration in Taiwan.

**Figure 1:**
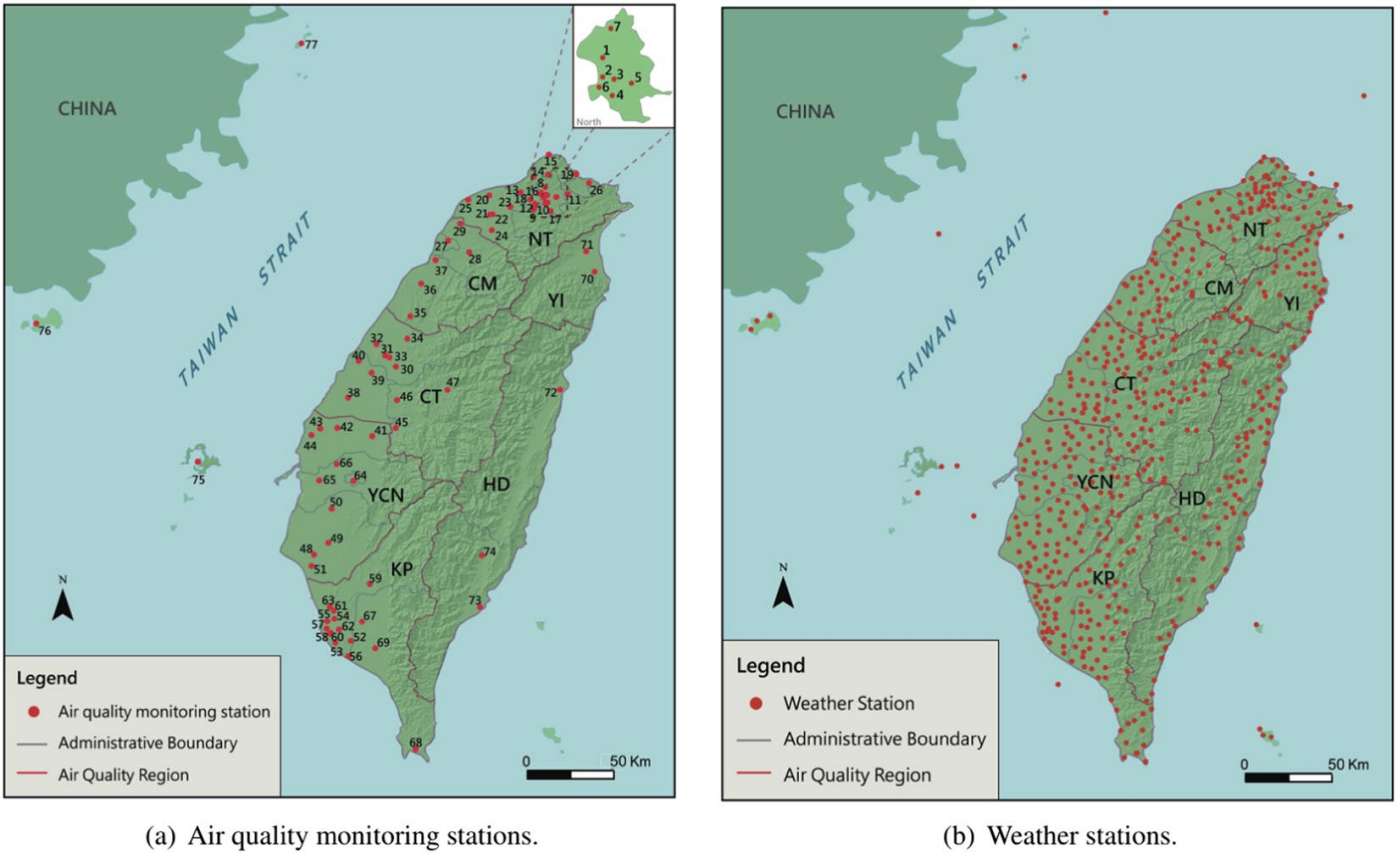
The (**a**) 77 air quality monitoring stations and (**b**) 580 weather stations set up across the seven air quality regions in Taiwan.

The large-scale database used in this study was built by the official EPA and Central Weather Bureau (CWB). The database contains more than 260,000 samples taken in 2017 from 77 air monitoring stations (from the EPA) and 580 weather stations (from the CWB) in Taiwan. Fig. 1(a) displays the geographical positions of the 77 air stations. The map indicates that Taiwan island is surrounded by the ocean and has the Central Mountain Range (CMR) running from the north to the south. The complex terrain and different meteorological conditions create spatial and seasonal variations in the PM_2.5_ concentration in Taiwan. Considering the different meteorological and geographical conditions, Taiwan has been divided into seven regions by the EAP for regional air quality monitoring. As illustrated in Fig. 1(a), the seven regions are northern Taiwan (NT), Chu-Miao (CM) area, central Taiwan (CT), Yun-Chia-Nan (YCN) area, Kao-Ping (KP) area, Hua-Dong (HD) area, and Yilan (YI)^30^. In addition to the geography, PM_2.5_ is also heavily influenced by the weather conditions. To offer accurate weather conditions and predictions, the CWB has set up 580 weather stations in Taiwan, as depicted in Fig. 1(b).

**Figure 2:**
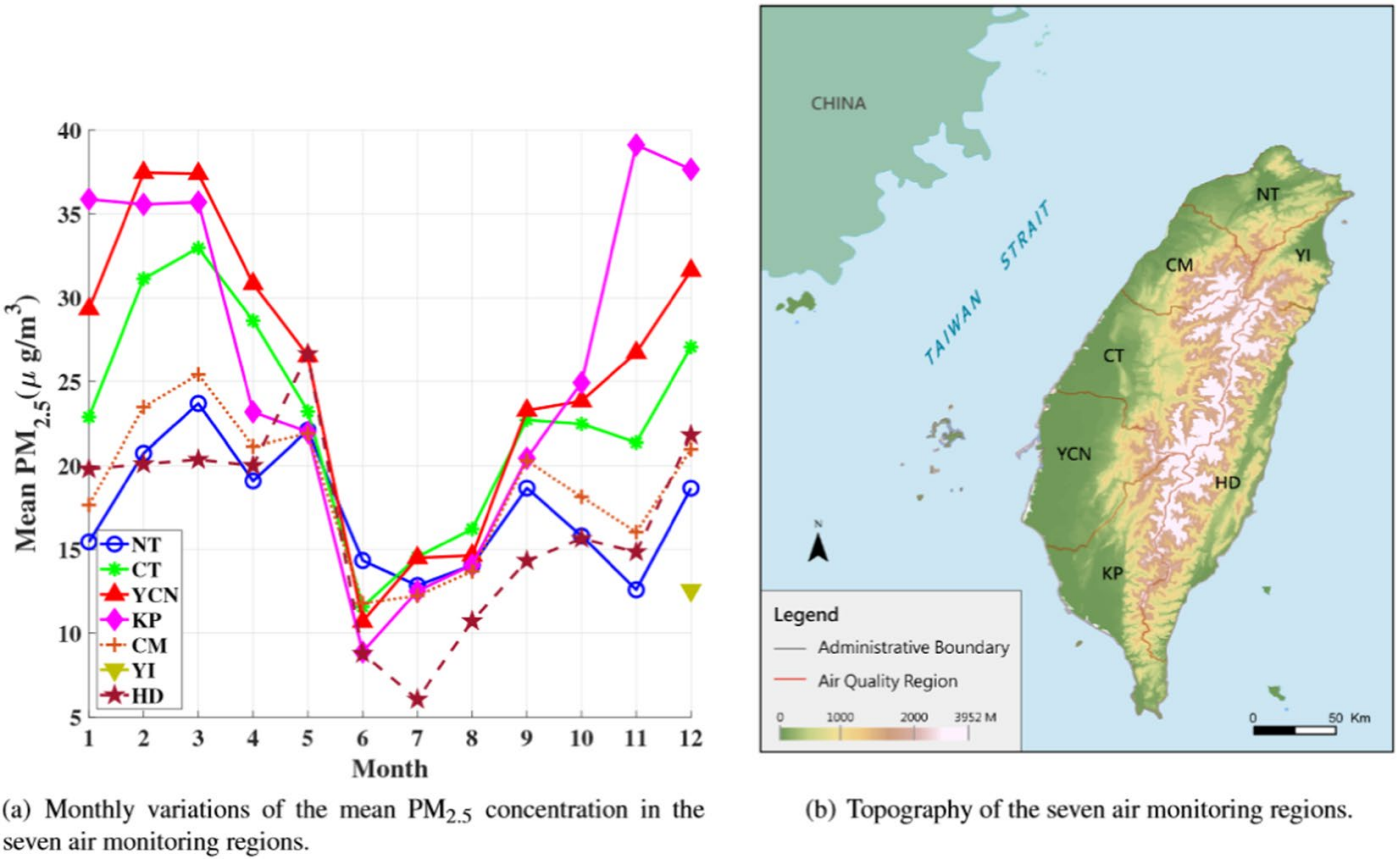
(**a**) Monthly variations of the mean PM_2.5_ concentration in the seven air monitoring regions and (**b**) their topography.

Figure 2 illustrates the monthly temporal variation of the mean PM_2.5_ concentration in the seven regions, where YI has missing data except for December. This figure indicates that the PM_2.5_ concentration reaches a peak in most regions during spring (February, March, and April), drops dramatically during summer (May, June, and July), and starts to go upwards during autumn (September, October, and November) and winter (November, December, and January).

Starting from autumn (September, October, and November), the prevailing wind (NEM) affects the northern part of the island, thereby easily dispersing and diluting air pollutants. The NEM becomes strong during the winter; however, it also brings LRT pollutants from China, which affect the air quality. Thus, air pollution is severe because the NEM is blocked by CMR; therefore, the southern part of Taiwan becomes a lee side region and forms stagnant wind conditions that increase the accumulation of air pollutants^30,31^. By contrast, during spring a stable weather system without strong winds is observed, especially in the northern part of the island^29^. Besides, the boundary layer height is also one of the factors for decreasing PM_2.5_ during summer. On the contrary, during autumn and winter, temperature inversion limits the boundary layer height and results in increasing PM_2.5_ concentration. Figure 2 also indicates that Taiwan has superior air quality during the summer because the prevailing wind (SWM) easily disperses and dilutes air pollutants. Moreover, the subtropical high pressure over the Pacific Ocean in the summer causes high convection of air mass, which leads to the vertical dispersion of pollutants.

After examining the temporal variations, we investigate the geographical divergence. Fig. 2 indicates that YCN and KP have the worst air quality. According to the EPA standards, when the PM_2.5_ concentration is larger than 35.4 *μg*/*m*^3^, the air quality could cause health problems to citizens. From Fig. 2, the YCN and KP regions exceed the threshold values for 2 and 5 months, respectively. By contrast, NT and HD have the best air quality because NT is a financial and business center and HD is an undeveloped green space. As expected, these two regions have only minor industrial pollution. In Taiwan, HD is the most livable place with good air quality because the mean PM_2.5_ concentration is below 15.4 *μg*/*m*^3^ for several months.

An interesting region is CT, where the mean PM_2.5_ concentration is lower than that in only KP and YCN. The central part of Taiwan is subject to both meteorological conditions and topographic effects because most residents live in the basin area surrounded by hills and mountains. Furthermore, the second-largest coal-fired power station in the world, that is, the Taichung Power Plant, is located in this region. The power plant accounts for 15% of Taichung City’s PM_2.5_ concentration^32^.

**Figure 3:**
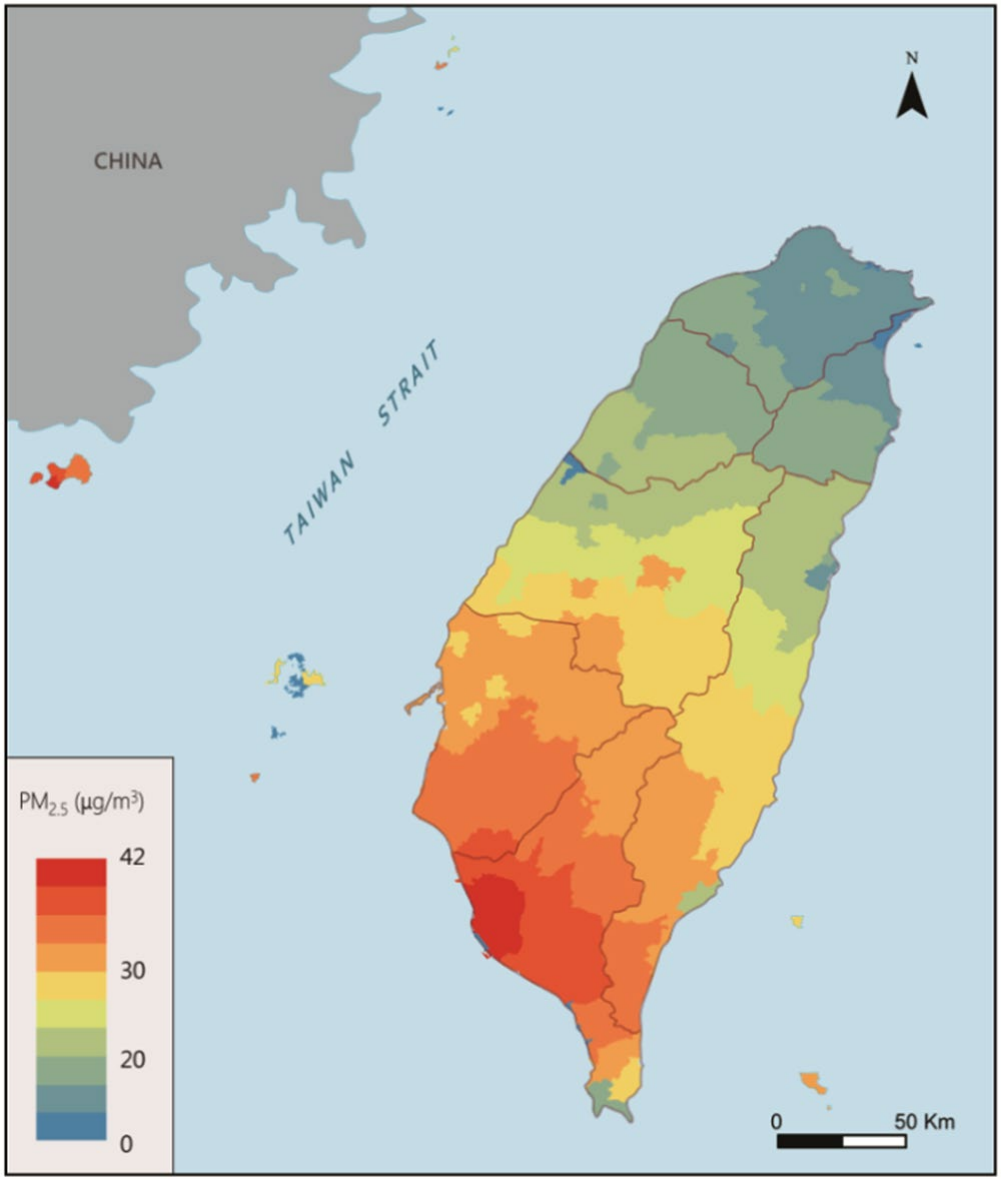
Estimate of the PM_2.5_ concentration of Taiwan in December.

  Figure 3 shows the estimate of the PM_2.5_ concentration of Taiwan in December using IDW (inverse distance weighted) technique, which is a interpolation method via spatial average for unseen locations^33,34^. This figure shows that NEM is strong in December, resulting in better air quality in the northern Taiwan. However, the NEM is blocked by CMR. This results in weak wind in the southern Taiwan and bad atmospheric diffusion condition.

## Results

The data provided by the EPA and CWB for 2017 were collected and processed. These data contained more than 260,000 samples covering 264,799 h and 36 attributes. In this study, 81 independent parameters were selected from each air station and its four nearest weather stations as features, and gradient boosting with regression was used as the learning model to forecast the PM_2.5_ concentration for the next 24 h. The feature extraction process and learning algorithm are described in detail in the following sections. This study used five-fold cross validation to justify the prediction results. The entire data were randomly split into five folds, of which four were used for training and one was used for testing.

## PM_2.5_ Prediction Results

**Figure 4:**
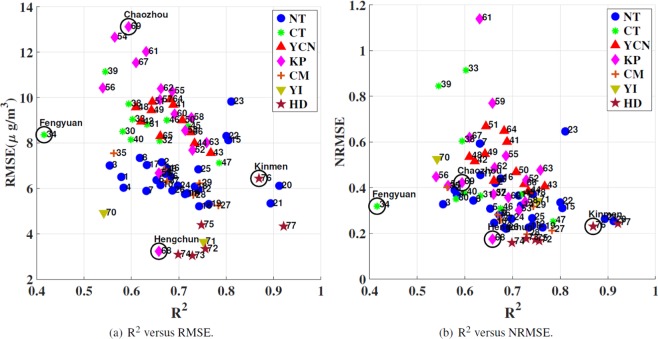
Annual average results of PM_2.5_ prediction performance for the 77 air stations in Taiwan.

The RMSE, normalized RMSE (NRMSE), and R^2^ were used as prediction performance metrics in the experiments. The RMSE indicates the mean fluctuation between the observed and predicted values. A smaller RMSE and NRMSE indicate a superior forecasting performance. R^2^ measures the proportion of the variance of observed values that is predictable in the case of multiple regression. When R^2^ = 1, the observed values are perfectly predicted. Fig. 4 displays the annual average results of forecasting performance of 77 air stations in the seven regions of Taiwan. The results in Fig. 4(a) generally agree with the geographical divergence. Most stations in the YCN, KP, and CT regions are distributed in the upper left. Furthermore, we can observe that the similarity between Fig. 4(a,b) lies in some boundary stations including stations 34, 68, and 76. Although the station 61 shows the highest NRMSE instead of station 69, both stations show the similar RMSE, as shown in Fig. 4(a). In addition, some trend is consistent in Fig. 4(a,b) such as the distribution of HD regions (the lower right), CT regions (the upper left), and NT regions (middle). Next, the separation between regions obviously reduced due to the normalization. For example, YCN and NT are clearly separated in Fig. 4(a) whereas they are mixed in Fig. 4(b). Using NRMSE, it is also difficult to distinguish the KP regions among the middle ranges. Thus, each performance metric has its own advantage and dis-advantages. NRMSE provides fair prediction performance comparison among each monitoring stations while RMSE makes the comparison of air quality regions easily due to higher separation ability. In addition to the large RMSE and NRMSE, the small R^2^ values imply that these regions may contain an increased number of hidden factors that may influence the prediction model. Thus, an interesting observation is that although YCN and KP have worse air quality than CT, the R^2^ value of CT is lower than that of YCN and KP. The R^2^ value for station 34 (Fengyuan station in CT) is only 0.41, and is the worst among all stations. This issue is discussed in detail in the following paragraph. By contrast, most stations in the HD, YI, and NT regions are distributed in the lower right. For stations 20, 21, 76, and 77, R^2^ is approximately equal to 0.9, which indicates that the forecasting model works nearly perfectly for these stations. At some stations, the RMSE values are smaller than 4 and the NRMSE values are smaller than 0.2, which indicates that the prediction value approaches the ground truth. Among these stations, we observe that although station 68 (Hengchuen) belongs to the KP region, its performance is completely different from that of other stations in this region. The reason for this observation may be that station 68 is located in a national park in south Taiwan, where few anthropic activities occur, which results in a low RMSE and NRMSE.

**Figure 5:**
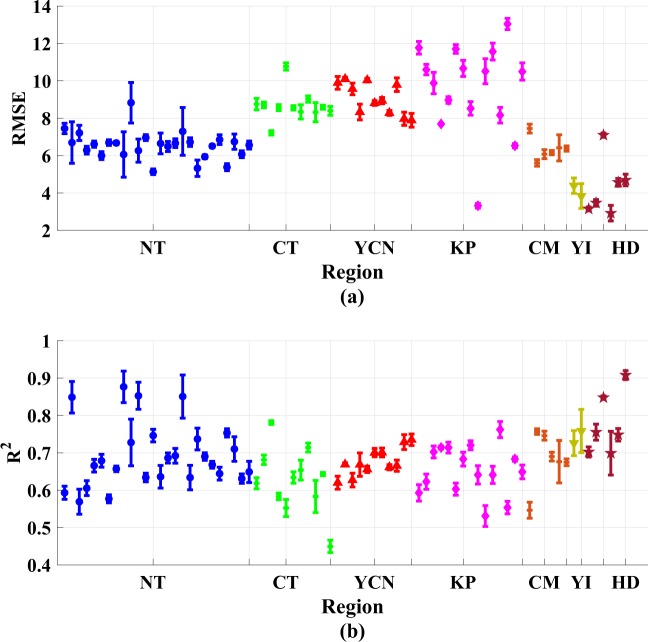
STD of (**a**) RMSE and (**b**) R^2^ across 5-folds validations in seven regions.

  Figure 5 shows the standard deviations (STD) of RMSE and R^2^ across 5-folds validations. Besides, we can observe that the maximum STD of RMSE is less than 1 and that of R^2^ is less than 0.1. In addition, this figure also indicates that almost all monitoring stations show the similar and stable STD, while there are only minor stations show relatively higher STD in the NT regions. Thus, the forecast models are not overfitting for the air monitoring stations in Taiwan during the experiments.

**Figure 6:**
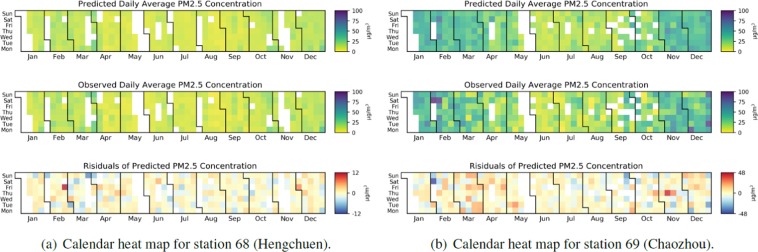
Comparison between the observed and predicted PM_2.5_ values for 2017 between (**a**) station 68 (Hengchuen) and (**b**) station 69 (Chaozhou).

  Figure 6 displays calendar heat maps of predicted, observed, and residual values at station 68 (Hengchuen) and 69 (Chaozhou). In Fig. 6(a), Hengchuen exhibits small residuals in the PM_2.5_ concentration over the year, a low PM_2.5_ concentration (maximum is only approximately 30 *μg*∕*m*^3^), and minor differences between the observed and predicted PM_2.5_ values. By contrast, as displayed in Fig. 6(b), Chaozhou exhibits large residuals in the PM_2.5_ concentration over the year, a high PM_2.5_ concentration (maximum is approximately 100 *μg*∕*m*^3^), and significant differences between the observed and predicted PM_2.5_ values. Besides, Fig. 6(b) illustrates the higher observed values in winter (November, December, and January) and spring (February, March, and April) at Chaozhou. The results indicate that although both Hengchuen and Chaozhou are in the KP region, their forecasting performance is significantly different. Hengchuen is located in a national park, where Chaozhou is very close to the Linyuan industrial area. Therefore, in addition to the between-region divergence, the in-region difference is also an important issue when considering the prediction performance of individual air monitoring stations.

## Comparison Between Taiwan and London

We applied another public air quality database to validate the aforementioned study results. Recently, the air quality data for London and southeast England was made available for independent scientific measurements and assessment^35,36^. Therefore, we selected London as a benchmark region for comparison. By using the same analysis procedure as that for Taiwan, experiments were conducted to collect hourly data from air and weather stations for 6 years (2012–2018) in London to provide a fair comparison of air quality prediction between different countries^37^.

**Figure 7:**
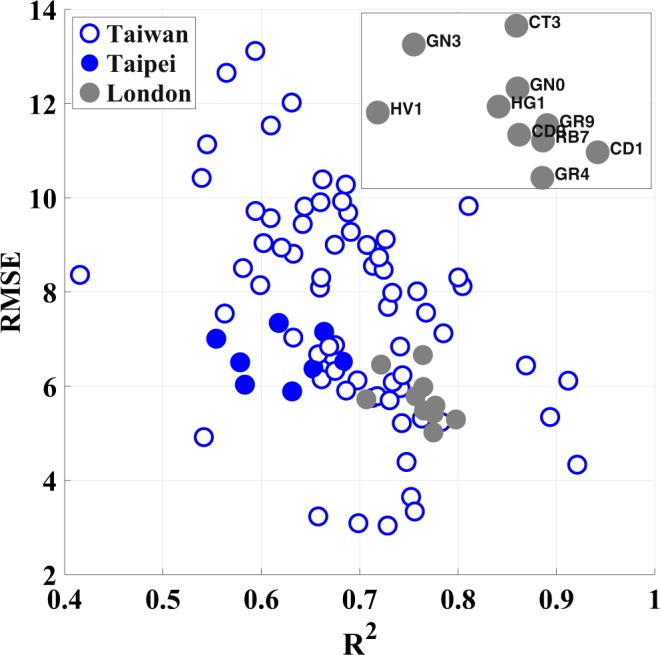
Comparison of the forecasting performance between Taiwan, Taipei, and London.

  Figure 7 illustrates the comparison results, where the empty blue circles and solid gray circles represent air stations in Taiwan and London, respectively. The subfigure depicts the index abbreviations of air stations in London^35^. Taiwan has 77 air stations for detecting PM_2.5_ concentration, whereas London has only 11 ones. The full names, acronyms, and environments of London stations are shown in Table 1. whereas that in Taiwan is 0.4–0.9. The RMSE results exhibit the same tendency. The RMSE in London is 5–7, whereas that in Taiwan is 3–13. These results indicate that the air quality forecasting performance in London is significantly superior to that in Taiwan even when using the same algorithm. This may be attributed to three main factors. First, from the perspective of geography, London is located in a basin, whereas Taiwan has considerably complicated terrain. Second, London is a financial center without domestic pollution sources. By contrast, most pollution in Taiwan originates from domestic sources, such as in KP and YCN. Third, Taiwan has transboundary issues because air pollution may be transported from China. These factors explain why London significantly outperforms Taiwan and also indicate that dedicated air quality forecasting in Taiwan is challenging.

**Table 1:** The acronyms for the London stations.

Index	SiteCode	SiteName	SiteType	DataOwner
1	CD1	Camden - Swiss Cottage	Kerbside	Camden
2	CT3	City of London - Sir John Cass School	Urban Background	City of London
3	GN0	Greenwich - A206 Burrage Grove	Roadside	Greenwich
4	GN3	Greenwich - Plumstead High Street	Roadside	Greenwich
5	GR4	Greenwich - Eltham	Suburban	Greenwich
6	GR9	Greenwich - Westhorne Avenue	Roadside	Greenwich
7	HV1	Havering - Rainham	Roadside	Havering
8	LW2	Lewisham - New Cross	Roadside	Lewisham
9	RB7	Redbridge - Ley Street	Urban Background	Redbridge
10	TD5	Richmond Upon Thames - Bushy Park	Suburban	Richmond
11	TH4	Tower Hamlets - Blackwall	Roadside	Tower

Next, we selected stations in the Taipei region in Taiwan (solid blue circles in Fig. 7) for comparing with stations in London for two reasons. First, most northern stations are located in Taipei, which lies in a basin. In addition, Taipei is a financial center without domestic pollution sources. Thus, Taipei is similar to London in terms of the topography, pollution sources, and number of air stations. The comparison in Fig. 7 indicates that Taipei has similar prediction accuracy compared with that of London from the viewpoint of both the RMSE and R^2^ values. Fig. 7 indicates that the RMSE in Taiwan is 3–13 and that in Taipei is 5–8. In other words, PM_2.5_ prediction in Taipei is more accurate than that at other stations in Taiwan. The results obtained from an alternative database for London verified the advantages of the proposed prediction mechanism. If a city has a pure topography (basin) and zero domestic pollution sources (e.g., Taipei and London), the PM_2.5_ prediction performance is expected to be stationary and reliable with a RMSE of approximately 6 and an R^2^ value of 0.75.

## Effect of Adding Industry-Related CEMS Features

In recent years, air pollution has worsened in CT, especially in Taichung City, for reasons that remain unclear. Domestic pollution could be one of the reasons because the coal-fired power station in Taichung increased power generation in 2017. The power generation was increased at the directive of the Taiwan government to satisfy future energy requirement and to promote the nuclear-free homeland policy. The results indicate that the forecasting performance is poor in CT, as displayed in Fig. 4. In particular, station 34 has the lowest R^2^ value. This result is consistent with the coal-fired power station’s policy for increasing power generation. We explored the possibility of using additional industry-related features to achieve an enhanced prediction performance. The effect of adding industry-related features may also provide some scientific evidence for the distribution of pollution sources.

The question is whether improving the prediction accuracy is possible when considering industry-related information. To answer this question, we used the alternative CEMS database to investigate the effect of adding industry-related features on the PM_2.5_ prediction. The emission of factory gas through chimneys has been managed and monitored by the CEMS of the EPA since 1993. CEMS detects several parameters, including the CO, SO_2_, O_2_, nitrogen oxides, and hydrogen chloride concentrations; temperature; opacity, and emission flow rate^38^. Besides, wind speed and direction are applied for considering diffusion of the detected items. CEMS has collaborated with the EPA since 2003, and the data has recently been made publicly available for academic research.

**Figure 8:**
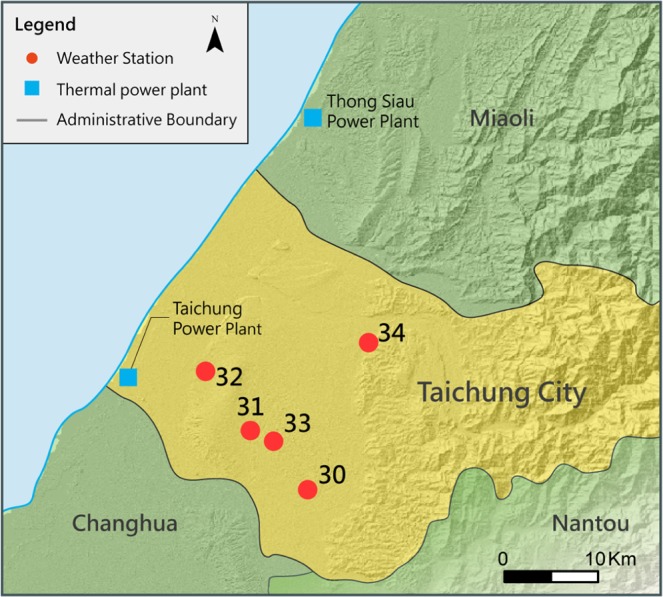
Geographical positions of the air stations and power plants in Taichung.

  Figure 8 displays the geographical positions of Taichung’s coal-fired power plants and air stations 30-34 in Taichung City, which are indicated by the yellow area in the map. In addition to the large coal-fired power station, Fig. 8 also shows the geographical position of the Thong-Siau gas-fired power plants, which is the second-largest station in CT and is close to the air stations in Taichung City. Therefore, considering these two thermal power plants when making PM_2.5_ predictions in Taichung is reasonable.

**Figure 9:**
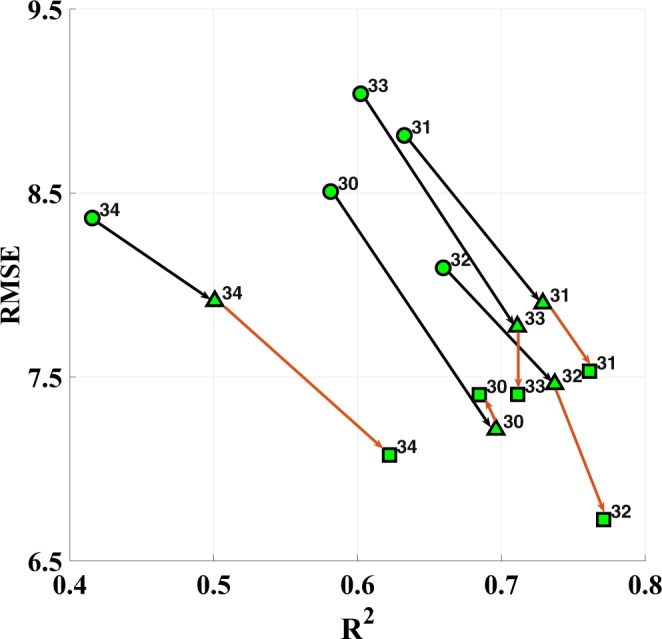
CEMS impact of the Taichung and Thong-Siau power plants on five air monitoring stations in Taichung City, where the circles represent the performance without CEMS, triangles represent the impact of adding Taichung Power Plant’s attributes, and squares represent the impact of adding features from both the Taichung and Thong-Siau power plants.

  Figure 9 illustrates the effect of adding industry-related features in Taichung, in which all the CEMS data were included as features in the training model. The figure indicates that after considering the CEMS, the forecast performance significantly improves in terms of the RMSE and R^2^ values. In general, the highest improvement is observed when adding Taichung Power Plant’s attributes. Upon also adding Thong-Siau power plant’s features, stations 34 and 32 exhibit a similar performance, stations 31 and 33 exhibit marginal improvement in the performance, and station 30 exhibit a marginal decrease in the performance.

An interesting observation is that the improvement in the forecast performance is highly related to the distance between the power plants and the air stations. For example, stations 30-33 are closer to Taichung Power Plant than station 34, as shown in Fig 8. This may explain why the improvement upon adding Taichung Power Plant is the most insignificant at station 34 compared with the other air stations. Besides, station 34 is located near CMR and has higher altitude than nearby stations. It has been influenced by both the Taichung and Thong-Siau power plants and suffers from high PM_2.5_ value due to bad atmospheric diffusion conditions. Furthermore, station 34 is close to Thong-Siau power station plant, and the improvement upon adding Thong-Siau power plant’s features is the largest among all the air stations. Similarly, station 30 is the farthest from the Thong-Siau power plant. This may explain why the forecast performance decreases marginally after considering the features of Thong-Siau power plant. In summary, the R^2^ values increase by 0.13, whereas the RMSE decreases by 1.50 on average (Fig. 9). These results indicate that the PM_2.5_ prediction performance in Taichung improves significantly on considering the neighboring coal-fired power plants.

**Figure 10:**
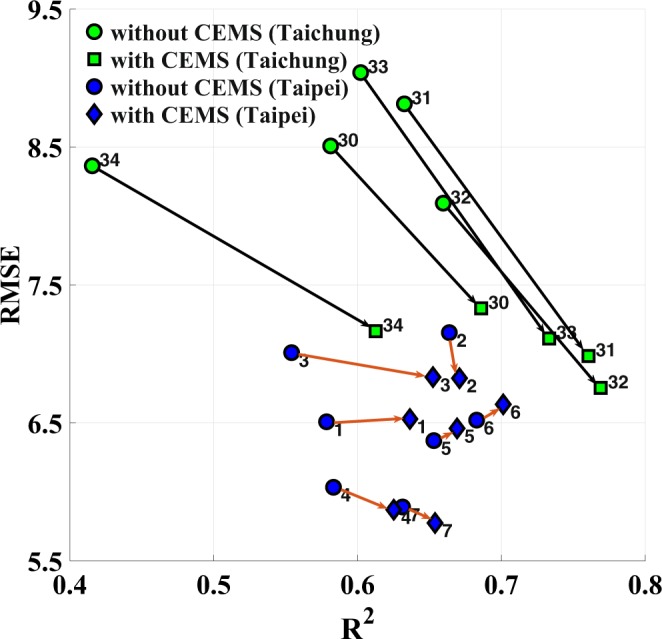
Comparison of the CEMS impact between Taichung and Taipei. The prediction results of Taipei with and without CEMS are represented by blue solid circles and diamonds, respectively. The prediction results of Taichung (shown in Fig. 9) with and without CEMS are represented by green solid circles and squares, respectively, for a comparison.

Next, we follow the same procedure to determine whether the same trend can be obtained in Taipei City. Although no industrial power plants exist in Taipei, the CEMS data, including data from garbage incinerators at Mucha, Neihu, and Beitou, were applied as alternative industry-related features to determine the performance improvement for air stations in Taipei. Figure 10 depicts the comparison between Taichung and Taipei. This figure indicates that although adding CEMS features still provides a marginal performance improvement in Taipei, the degree of improvement is not comparable to that in Taichung. Industrial pollution appears to have a stronger impact in Taichung than in Taipei, especially in terms of the PM_2.5_ prediction. This observation fits recent developments of Taipei and Taichung. Taipei City is a financial and business center in Taiwan with minor industrial impact, whereas Taichung City experiences increased air pollution due to coal-fired power plants.

## Discussion

The air quality forecast has aroused attention from governments and scientists for improving environmental quality of citizens and upgrading environmental sensing studies. Because numerous scientific studies have linked PM_2.5_ (particulate matter) to health problems, this study proposes a machine learning approach based on GBM to predict the PM_2.5_ concentration in Taiwan.

By extracting temporal sequences from air monitoring and weather stations into features, the proposed mechanism efficiently predicts the air quality in terms of the PM_2.5_ concentration for the next 24 h at individual air monitoring stations. By performing experiments over 1 year, this study investigated the geographical and meteorological divergence of forecasting results and measurements at seven regional monitoring areas in Taiwan. The results from the alternative London database verified the proposed prediction mechanism. We observe that Taiwan has a more complex environment than London due to domestic pollution sources, overseas pollution sources (mainly from China), and geographic topography; however, Taipei exhibits a similar prediction performance to London. This may be because these two cities have similar topography (basin) and are financial centers without domestic pollution sources. Finally, because domestic industrial pollution makes prediction difficult, this study proposes an enhanced fusion version of the prediction model by incorporating additional features from the CEMS to improve the prediction accuracy. After considering the industrial impact, the proposed method achieves significant improvement in the R^2^ values (0.58–0.71 on average) and considerably decreases the RMSE (8.56–7.06 on average)compared with the conventional approach for Taichung City. In the future, we will try to use recent advanced dynamic neural networks to improve the performance. We will also investigate deeper and more complex model structure when more training data become available for air quality prediction.

## Methods

### Data collection

The EPA, CWB, and CEMS databases are the main data sources used to forecast air quality. EPA uses an automatic continuous monitoring system to immediately identify and respond to uncommom emerging signals. This system collects air quality data every hour^39,40^. EPA provided more than 260,000 samples from 77 air stations in 2017. The measured attributes include the index, city, county, station name, date, detected items, and time in hours. The detected items include PM_2.5_, NO_2_, PM_10_, NO, NO_*X*_, SO_2_, CO, O_3_, THC, NMHC, and CH_4_. Specifically, PM_2.5_ represents particulate matter with a diameter less than 2.5 *μm*, and NO_2_ is one of a group of highly reactive gases. These substances are primarily released into the air from the burning of fuel in cars, trucks buses, and power plants.

Taiwan’s EPA has strict regulations and guidelines in regards to quality assurance operations for air quality monitoring to achieve the EPA’s data quality objectives (DQO). Every year the EPA has to perform quality assurance operations for the air quality monitoring system in order to evaluate the accuracy of air quality measurements. Those data were recorded in EPA’s quality assurance (QA) operations annual report of air quality monitoring system. For our PM25 data (2017), the accuracy was 96.3%. The annual report from 2001 to 2017 can be downloaded at the EPA website^41^. In Taiwan, the stations are categorized by the EPA as 6 types, which may give a general idea about the location of the stations:

- 60 general stations. Most of the stations are located in urban and suburban area.
- 5 industrial stations. All of the stations are close to industrial factories.
- 5 traffic stations. These stations are located aside the main roads and have lower sampling altitude.
- 4 background stations (2 stations simultaneous as the general station). These stations are away from pollution sources and provide the baseline monitoring data.
- 2 national park stations (1 station simultaneous as the general station).
- 2 other air quality monitoring stations. These two stations are located in rural area.

CWB has set up 580 weather stations to monitor and report weather conditions in Taiwan^42^. The attributes of weather information include longitude, latitude, station name, city, county, wind speed, wind direction, temperature, and pressure. The number and date of datasets from the CWB and EPA must be synchronized for model training. The CEMS was established by the EPA to monitor the gases emitted from chimneys of industries since 1993. The detected items include the CO, SO_2_, O_2_, nitrogen oxides, and hydrogen chloride concentrations; temperature; opacity; and total emission flow rate. Specifically, emission flow rate indicates total emission materials from a chimney and its unit is cubic meter per hour^38^. The data has been recently made publicly available for academic research, and we used this data for training to study the influence of the CEMS on Taichung and Taipei.

### Feature extraction

We derived and extracted features from the EPA and CWB hourly data for model training. There exist 81 features from each air station and its four nearest weather stations. Specifically, 21 features were identified from individual air stations and 15 were identified from a weather station. Tables 2 and 3 list the details of the 81 features for generating the GBM-based PM_2.5_ prediction model.

**Table 2:** Input features obtained from an air monitoring station.

Day	Air Monitoring Station	Dimensions
	Features	
Predicting day	Holiday or not, weekend or not, Saturday or not, Sunday or not, concentration difference, extrapolation of concentration, average pressure, average temperature, hour of the day, day of the week, and which year	11
Day before the predicting day Days before the predicting day	Holiday or not, weekend or not, Saturday or not, and Sunday or not, Concentration of air pollutant	5 × 2

**Table 3:** Input features from a weather station. The features from four weather stations and one air station are required for model training.

Day	Weather Station	Dimensions
	Features	
Predicting day Day before the predicting day Two days before the predicting day	pressure, temperature, wind speed, eastern wind speed, northern wind speed	5 × 3

When we forecast air pollution on a given day, the previous two days are also considered due to the memory effect. Furthermore, the difference between these two days’ PM_2.5_ concentration is defined as “concentration difference” In addition, because traffic flow strongly influences the air quality, the features consider whether a given day is regular day, weekend, or holiday. An air station’s weather conditions are represented by the mean pressure and mean temperature of nearby weather stations. Features in the training model also include the hour of day, day of week, and year to learn the trend and period of the temporal index. In summary, 21 features are extracted from an air station, as presented in Table 2.

We derived weather stations’ pressure, temperature, and wind speed from the features to represent the weather conditions at nearby air stations. Because wind can blow from any direction, the wind was split into the northern and eastern directions for simplicity. In summary, 15 features were inferred from each weather station, as presented in Table 3. Because the 580 weather stations are uniform distributed in Taiwan, neighboring weather stations should be enough for representing weather condition of each air quality station. However, number of weather stations may be varying according to the environments and validation tests. To provide a fair comparison among stations, we fixed the number as 4 in the experimental setup. Therefore, we used one air station and four neighboring weather stations to generate 81-dimensional feature vectors for model learning and PM_2.5_ forecasting.

### GBM

GBM combines fitting functions, loss functions, a decision tree, and gradient descent analysis^24^. The decision tree produces initial values for the fitting function with multiple regression, which deals with the many input variables considered in this study. Then, errors between the observed data sets and output values are calculated using a loss function. The frequently used loss functions include square-error, absolute-error, and negative binomial log-likelihood functions^43,44^. Thereafter, gradient descent analysis is applied to find the fitting function whose expected value of loss function is minimized. The aforementioned procedure is repeated to acquire the optimized fitting function.

The one-year data were randomly split into five folds, of which four were used for training models and one was used for testing models. Besides, the period of training data is chosen to cover a whole year to reduce seasonal changes when training prediction models. For training models, 31 features of the predicting day, 25 features of one day before predicting day, and 25 features of two days before predicting day comprise 81-dimensional input vector ***x***_*t*_, as shown in Tables 2 and 3, where *t* is the time index in hour. Specifically, the predicting day’s meteorological features are collected form weather forecast of the CWB. Because the target is to predict the next-24 hours PM_2.5_, the output is one-dimensional variable denoted as *y*_*t*+24_.

After *N* pairs of the input vector ***x***_*t*_ and the output variable *y*_*t*+24_ are given, a fitting function *F*(***x***_*t*_) is selected from unknown functions $F({{\boldsymbol{x}}}_{t},\beta {\prime} )$ produced by the decision tree. In addition, $\beta {\prime}$ is a gradient decent step size and $({{\boldsymbol{x}}}_{t}^{i},{y}_{t+24}^{i})$ is the *i*-th training sample pair. When the value of the loss function $L({y}_{t+24},F({{\boldsymbol{x}}}_{t},\beta {\prime} ))$ is minimized as

1 $$\beta =\arg \ {\min }_{\beta {\prime} }{\sum }_{i=1}^{N}L\left({y}_{t+24}^{i},F({{\boldsymbol{x}}}_{t}^{i},\beta {\prime} )\right),$$

the target function *F*(***x***_*t*_) is chosen to be *F*(***x***_*t*_, *β*). Note that in this study, *N* is 211840 since we collected one-year data and 80% of them were selected for training model. Besides, the gradient descent analysis is applied for optimized fitting function *F*(***x***_*t*_). The detailed process is described as follows. At the first step, initial guess function ${F}_{0}({{\boldsymbol{x}}}_{t},\beta {\prime} )$ is generated and initial gradient descent step size *β*_0_ is

2 $${\beta }_{0}=\arg \ {\min }_{\beta {\prime} }\mathop{\sum }\limits_{i=1}^{N}L({y}_{t+24}^{i},{F}_{0}({{\boldsymbol{x}}}_{t}^{i},\beta {\prime} ))$$

3 $${F}_{0}({{\boldsymbol{x}}}_{t})={F}_{0}({{\boldsymbol{x}}}_{t},{\beta }_{0}).$$

Then, we take gradient of loss function as a first-step base learner function *f*_1_(***x***_*t*_) as

4 $${f}_{1}({{\boldsymbol{x}}}_{t})=-{\nabla }_{{F}_{0}}L({y}_{t+24},{F}_{0}({{\boldsymbol{x}}}_{t})),$$

5 $${\beta }_{1}=\arg \,{\min }_{\beta {\prime} }\mathop{\sum }\limits_{i=1}^{N}L({{y}^{i}}_{t+24},[{F}_{0}({{\boldsymbol{x}}}_{t}^{i})+\beta {\prime} {f}_{1}({{\boldsymbol{x}}}_{t}^{i})]).$$

After *M* iterations, the target function *F*(***x***_*t*_) is expressed as

6 $$F({{\boldsymbol{x}}}_{t})={F}_{0}({{\boldsymbol{x}}}_{t})+\mathop{\sum }\limits_{m=1}^{M}{\beta }_{m}{f}_{m}({{\boldsymbol{x}}}_{t}),$$

where *f*_*m*_(***x***_*t*_) and *β*_*m*_ are expressed as follows

7 $${f}_{m}({{\boldsymbol{x}}}_{t})=-{\nabla }_{{F}_{m-1}}L({{\boldsymbol{y}}}_{t+24},{F}_{m-1}({{\boldsymbol{x}}}_{t}))$$

8 $${\beta }_{m}=\arg \ {\min }_{\beta {\prime} }\mathop{\sum }\limits_{i=1}^{N}L({{y}^{i}}_{t+24},[{F}_{m-1}({{\boldsymbol{x}}}_{t}^{i})+\beta {\prime} {f}_{m}({{\boldsymbol{x}}}_{t}^{i})]).$$

Finally, *F*(***x***_*t*_) is the GB-based prediction model. Then, during the online stage, the testing samples are put into the model to calculate the forecasting results. The entire GBM procedure is described in Algorithm 1.

**Algorithm 1:**
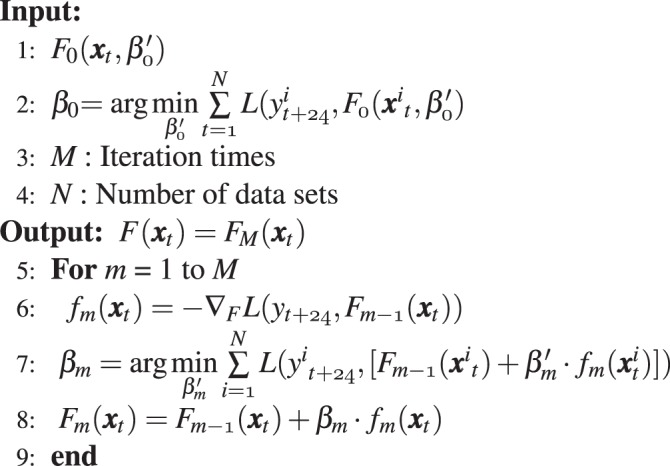
GBM.

## Data Availability

The air quality data and the weather condition data are available in the websites of https://taqm.epa.gov.tw/taqm/tw/YearlyDataDownload.aspx/EPA and http://farmer.iyard.org/cwb/cwb.htmCWB.
